# Association between initial prescribed minute ventilation and post-resuscitation partial pressure of arterial carbon dioxide in patients with post-cardiac arrest syndrome

**DOI:** 10.1186/2110-5820-4-9

**Published:** 2014-03-07

**Authors:** Brian W Roberts, J Hope Kilgannon, Michael E Chansky, Stephen Trzeciak

**Affiliations:** 1 The Department of Emergency Medicine, Cooper University Hospital, Cooper Medical School of Rowan University, One Cooper Plaza, K152, Camden, NJ 08103, USA; 2 The Department of Medicine, Division of Critical Care Medicine, Cooper University Hospital, Cooper Medical School of Rowan University, Camden, NJ, USA

**Keywords:** Cardiac arrest, Heart arrest, Cardiopulmonary resuscitation, Resuscitation, Anoxic brain injury, Shock, Hypocapnia, Hypercapnia, Minute ventilation

## Abstract

**Background:**

Post-cardiac arrest hypocapnia/hypercapnia have been associated with poor neurological outcome. However, the impact of arterial carbon dioxide (CO_2_) derangements during the immediate post-resuscitation period following cardiac arrest remains uncertain. We sought to test the correlation between prescribed minute ventilation and post-resuscitation partial pressure of CO_2_ (PaCO_2_), and to test the association between early PaCO_2_ and neurological outcome.

**Methods:**

We retrospectively analyzed a prospectively compiled single-center cardiac arrest registry. We included adult (age ≥ 18 years) patients who experienced a non-traumatic cardiac arrest and required mechanical ventilation. We analyzed initial post-resuscitation ventilator settings and initial arterial blood gas analysis (ABG) after initiation of post-resuscitation ventilator settings. We calculated prescribed minute ventilation:

$$\mathrm{MV}\;\left(\mathrm{mL}/\mathrm{kg}/\min\right)=\left[\left[\mathrm{tidal}\;\mathrm{volume}\;\left(\mathrm{TV}\right)/\mathrm{ideal}\;\mathrm{body}\;\mathrm{weight}\;\left(\mathrm{IBW}\right)\right]\;x\;\mathrm{respiratory}\;\mathrm{rate}\;\left(\mathrm{RR}\right)\right]$$

for each patient. We then used Pearson’s correlation to test the correlations between prescribed MV and PaCO_2_. We also determined whether patients had normocapnia (PaCO_2_ between 30 and 50 mmHg) on initial ABG and tested the association between normocapnia and good neurological function (Cerebral Performance Category 1 or 2) at hospital discharge using logistic regression analyses.

**Results:**

Seventy-five patients were included. The majority of patients were in-hospital arrests (85%). Pulseless electrical activity/asystole was the initial rhythm in 75% of patients. The median (IQR) TV, RR, and MV were 7 (7 to 8) mL/kg, 14 (14 to 16) breaths/minute, and 106 (91 to 125) mL/kg/min, respectively. Hypocapnia, normocapnia, and hypercapnia were found in 15%, 62%, and 23% of patients, respectively. Good neurological function occurred in 32% of all patients, and 18%, 43%, and 12% of patients with hypocapnia, normocapnia, and hypercapnia respectively. We found prescribed MV had only a weak correlation with initial PaCO_2_, R = -0.40 (*P* < 0.001). Normocapnia was associated with good neurological function, odds ratio 4.44 (95% CI 1.33 to 14.85).

**Conclusions:**

We found initial prescribed MV had only a weak correlation with subsequent PaCO_2_ and that early Normocapnia was associated with good neurological outcome. These data provide rationale for future research to determine the impact of PaCO_2_ management during mechanical ventilation in post-cardiac arrest patients.

## Background

After successful resuscitation from cardiac arrest, even with therapeutic hypothermia treatment [1-3], most patients either die in the hospital or suffer permanent, crippling neurological disability due to anoxic brain injury [4]. Finding new approaches to attenuate brain injury after return of spontaneous circulation (ROSC) is a high priority for resuscitation science. Partial pressure of arterial carbon dioxide (PaCO_2_) is a major regulator of cerebral blood flow. Hypocapnia and hypercapnia after ROSC have previously been demonstrated to be associated with poor outcomes in both adult and pediatric post-cardiac arrest patients [5,6]. In theory, hypocapnia could decrease cerebral blood flow, inducing or exacerbating cerebral ischemia [7-9], while hypercapnia could increase intracranial pressure and compound metabolic acidosis, which is common after ROSC [6,10-12]. Furthermore, among pediatric post-cardiac arrest patients, early exposure to PaCO_2_ derangements (that is on initial post-ROSC arterial blood gas (ABG)) was associated with in-hospital mortality, while exposure at 24 hours after ROSC was not associated [5], suggesting time sensitivity. There are important differences in the physiological and cardiac arrest characteristics of pediatric patients compared to adult patients. It is therefore unclear if early PaCO_2_ has the same association with neurological outcome in adult cardiac arrest patients.

Current American Heart Association (AHA) guidelines suggest an initial post-ROSC tidal volume (TV) of 6 to 8 mL/kg (based on ideal body weight (IBW)) followed by titration to a PaCO_2_ of 40 to 45 mmHg in patients resuscitated from cardiac arrest [13]. These guidelines go on to state there are no data on specific ventilation strategies in post-cardiac arrest syndrome. The current recommended approach can result in a time delay in optimizing PaCO_2_ (that is time to initial ABG data being available and subsequent ventilation adjustments). Although this period is usually brief, it is likely the time period when the injured brain is most susceptible to further damage. There is currently a paucity of data on the relationship between prescribed minute ventilation and early PaCO_2_ in post-cardiac arrest patients requiring mechanical ventilation. Determining this relationship is the first step towards developing a ventilation strategy aimed at more consistently achieving an optimal PaCO_2_ on the initial ABG after ROSC. Such a ventilation strategy could decrease the time to PaCO_2_ optimization in a greater proportion of patients.

We hypothesized initial post-resuscitation prescribed MV would have a weak correlation with the subsequent PaCO_2_. We also hypothesized that early PaCO_2_ would be associated with neurological outcome. The primary objective of this study was to test the correlation between initial post-resuscitation prescribed minute ventilation and subsequent PaCO_2_. Our secondary objective was to test if early PaCO_2_ (that is on initial ABG analysis after initiation of post-ROSC ventilation settings) was associated with neurological outcome.

## Methods

### Setting

We retrospectively analyzed a prospectively compiled and maintained cardiac arrest registry over a one year period (July 2010 to July 2011) at a single academic medical center, Cooper University Hospital in Camden, NJ, USA. We collected data pertaining to the index cardiac arrest event, and outcomes consistent with the Utstein style for reporting cardiac arrest research, including all post-ROSC variables recommended for post-resuscitation research [14,15]. In order to prospectively identify and capture data on consecutive post-cardiac arrest patients, we utilized a previously described 24-hour per day, 7-day per week paging system [6,16]. The Institutional Review Board approved this study with a waiver of written informed consent.

### Participants

We included both in- and out-of-hospital adult post-cardiac arrest patients who were mechanically ventilated after ROSC. We included adult (age ≥ 18 years) patients who experienced a non-traumatic cardiac arrest (defined as a documented absence of pulse and cardiopulmonary resuscitation (CPR) initiated) and required mechanical ventilation after ROSC. We excluded patients who died prior to an arterial blood gas analysis being obtained.

### Data collection

We abstracted the following variables: demographics, comorbidities, initial prescribed ventilator settings (that is TV, respiratory rate (RR), fraction of inspired oxygen, and positive end expiratory pressure) after ROSC, initial ABG analysis after initiation of post-ROSC ventilator settings, post-cardiac arrest interventions (for example, percutaneous coronary interventions and therapeutic hypothermia), and neurological status at hospital discharge (defined by the Cerebral Performance Category (CPC)). Arterial blood gas analysis was performed using the Siemens RAPIDLab 1265 (Erlangen, Germany) and pH-stat PaCO_2_ values (that is we analyzed PaCO_2_ at the patient’s actual temperature as opposed to warming the sample). We entered all data into a dedicated Access database (Microsoft Corporation, Redmond, WA, USA) and exported to StatPlus version 2009 (AnalystSoft Inc., Alexandria, VA, USA) for analysis.

### Outcome measures

The primary outcome was initial PaCO_2_ after initiation of post-ROSC ventilator settings. Secondary outcome was good neurological function at hospital discharge, defined as a CPC 1 or 2. The CPC is a validated five-point scale of neurological disability and historically the most commonly used outcome measure in post-cardiac arrest research (1: good cerebral performance, 2: moderate cerebral disability, 3: severe cerebral disability, 4: coma/vegetative state, 5: death) [14,17-19]. Patients with a CPC of 1 or 2 had sufficient cerebral function at discharge to live independently.

### Data analysis

We began the analysis with descriptive statistics. We displayed categorical data as counts and proportions. We described continuous data as median values and IQR or mean values and standard deviation, based on distribution of data. Since normal lung volumes are predicted based on sex and height [20,21], we calculated an IBW for each patient using the following formulae [22]:

$$\mathrm{Male}\;\mathrm{patients}:50+0.91\left(\mathrm{centimeters}\;\mathrm{of}\;\mathrm{height}-152.4\right)$$

$$\mathrm{Female}\;\mathrm{patients}:45.5+0.91\left(\mathrm{centimeters}\;\mathrm{of}\;\mathrm{height}-152.4\right)$$

We then calculated the initial prescribed MV (mL/kg/min) for each patient. MV was calculated as:

$$\mathrm{MV}\;\left(\mathrm{mL}/\mathrm{kg}/\min\right)=\left[\left(\mathrm{TV}/\mathrm{IBW}\right)\;\times \;\mathrm{prescribed}\;\mathrm{RR}\right]$$

We used Pearson’s linear correlation to test the correlation between prescribed MV and initial post-resuscitation PaCO_2_. We then used linear regression analysis to calculate R^2^, using prescribed MV as the independent variable and subsequent PaCO_2_ as the dependent variable.

For the purposes of additional analyses, we divided the cohort by initial PaCO_2_ after initiation of post-ROSC ventilator settings into hypocapnia, normocapnia, and hypercapnia (defined as PaCO_2_ ≤ 30, 31 to 49, and ≥ 50 mmHg, respectively, based on previously published cutoffs in studies of traumatic brain injury and post-cardiac arrest) [5,6,23]. We used univariable logistic regression to test the association between early normocapnia and good neurological function at hospital discharge. In order to ensure early normocapnia was independently associated with good neurological outcome, we performed sensitivity analyses adjusting for candidate variables known to be strong predictors of poor outcome in post-cardiac arrest patients. We selected the following candidate variables for the regression models: (1) initial cardiac rhythm (asystole or pulseless electrical activity (PEA) versus ventricular fibrillation/ventricular tachycardia (VF/VT)), (2) prolonged duration of cardiopulmonary resuscitation (CPR duration > 20 minutes) [4,24-29], (3) post-resuscitation shock (defined as systolic blood pressure < 100 mmHg or vasopressor support required to maintain systolic blood pressure > 100 mmHg during the first 24 hours after ROSC) [6,30,31], (4) metabolic acidosis (defined as one or more recorded base deficit ≤ -6 mmol/L during the first 24 hours after ROSC) [6,32], (5) age (decile), (6) pre-arrest comorbidities (that is Charlson comorbidities index) [33], (7) pre-arrest pulmonary disease, and (8) initiation of therapeutic hypothermia.

We also performed an additional multivariable logistic regression analysis to test the association between a narrower early PaCO_2_ range (between 35 and 45 mmHg) and good neurological function at hospital discharge.

## Results

One hundred and thirteen patients met all inclusion criteria. Thirty-eight patients died prior to ABG analysis being obtained. Of the 75 included patients, the majority of patients suffered in-hospital cardiac arrests with PEA/asystole as the initial rhythm (49/75 (65%)), and few patients were out-of-hospital cardiac arrest patients with pulseless VF/VT as the initial rhythm (4/75 (5%)). Therapeutic hypothermia was performed on 100% (4/4) of patients with out-of-hospital, VF/VT cardiac arrest (indicated population (that is Class I recommendation)) [13], and in 44% (33/75) of all patients. Coronary angiography was performed in 12/75 (16%) of patients and of these patients 7/12 (58%) underwent percutaneous coronary intervention. A greater proportion of patients had pre-arrest pulmonary disease among patients with hypercapnia compared to normocapnia, 47% versus 23% respectively. However, this difference was not found to be statistically significant (*P* = 0.07 using the Chi square test). Table 1 displays baseline data for all subjects in the cohort, as well as for patients with hypocapnia, normocapnia, and hypercapnia.

**Table 1:** Baseline data for all subjects at the time of cardiac arrest

	**All subjects**	**Hypocapnia**	**Normocapnia**	**Hypercapnia**
	**n = 75**	**n = 11**	**n = 47**	**n = 17**
Age (years (SD))	66 (16)	71 (16)	66 (16)	62 (15)
Female gender (n (%))	33 (44)	7 (64)	18 (38)	8 (47)
Pre-existing comorbidities (n (%))				
diabetes	33 (44)	4 (36)	23 (49)	6 (35)
known coronary artery disease	18 (24)	6 (55)	8 (17)	4 (24)
hypertension	44 (59)	9 (82)	29 (62)	6 (35)
malignancy	17 (23)	3 (27)	11 (23)	3 (18)
renal insufficiency	16 (21)	4 (36)	10 (21)	2 (12)
pulmonary disease	22 (29)	3 (27)	11 (23)	8 (47)
cerebral vascular disease	2 (3)	0	2 (4)	0
congestive heart failure	10 (13)	1 (9)	6 (13)	3 (18)
Charlson comorbidity score [33] (median (IQR))	2 (1 to 4)	3 (2 to 4)	2 (1 to 4)	2 (1 to 6)
Arrest location (n (%))				
Out-of-hospital	11 (15)	0	6 (13)	5 (29)
In-hospital	64 (85)	11 (100)	41 (87)	12 (71)
Initial arrest rhythm (n (%))				
PEA/asystole	56 (75)	7 (64)	35 (74)	14 (82)
VF/VT	19 (25)	4 (36)	12 (26)	3 (18)
CPR duration > 20 minutes (n (%))	9 (12)	0	4 (9)	5 (29)

CPR, cardiopulmonary resuscitation; PEA, pulseless electrical activity; VF, ventricular fibrillation; VT ventricular tachycardia.

The majority of patients were found to have post-resuscitation shock (69/75 (92%)), as well as a metabolic acidosis (49/75 (65%)) during the post-cardiac arrest period. Of note, pH was found to be lower among patients with hypercapnia compared to normocapnia 7.04 (6.98 to 7.13) versus 7.29 (7.15 to 7.37) respectively, (*P* = < 0.0001 using the Mann-Whitney *U*-test), while no difference was found in bicarbonate between patients with hypercapnia compared to normocapnia 18 (14 to 23) versus 19 (14 to 23) mmol/L respectively, (*P* = 0.91 using the Mann-Whitney *U*-test). A lower pH among patients with hypercapnia is expected secondary to respiratory acidosis. Table 2 displays post-cardiac arrest data for all subjects, as well as for patients with hypocapnia, normocapnia, and hypercapnia.

**Table 2:** Post-cardiac arrest data for all subjects (displayed as median (IQR) unless otherwise noted)

	**All subjects**	**Hypocapnia**	**Normocapnia**	**Hypercapnia**
	**n = 75**	**n = 11**	**n = 47**	**n = 17**
Initial prescribed ventilation Settings				
FiO_2_^‡^ (%)	100 (80 to 100)	100 (70 to 100)	100 (68 to 100)	100 (100 to 100)
PEEP (cmH_2_O)	5 (5 to 5)	5 (5 to 5)	5 (5 to 5)	5 (5 to 5)
Tidal Volume (mL/kg)	7 (7 to 8)	8 (7 to 8)	7 (7 to 8)	7 (6 to 7)
Respiratory rate (breaths/minute)	14 (14 to 16)	14 (14 to 21)	14 (14 to 16)	14 (12 to 14)
Post-resuscitation shock^a^ (n (%))	69 (92)	11 (100)	41 (87)	17 (100)
Metabolic acidosis^b^ (n (%))	49 (65)	9 (82)	27 (57)	13 (76)
pH	7.24	7.27	7.29	7.04
	(7.07 to 7.33)	(7.05 to 7.34)	(7.15 to 7.37)	(6.98 to 7.13)
Bicarbonate (mmol/L)	18 (13 to 23)	12 (7 to 18)	19 (14 to 23)	18 (14 to 23)

^a^Defined as systolic blood pressure < 100 mmHg or vasopressor support required to maintain systolic blood pressure > 100 mmHg during the first 24 hours after return of spontaneous circulation; ^b^Defined as a base deficit ≤ - 6 mmol/L during the first 24 hours after return of spontaneous circulation; ^‡^FiO_2_, fraction of inspired oxygen; PEEP, peak end expiratory pressure.

The majority of patients were prescribed pressure regulated volume control as the initial mode of ventilation (71/75 (95%)). Three patients (4%) were prescribed volume control and one patient (1%) was prescribed synchronized intermittent mandatory ventilation. The median and IQR TV, RR, and MV were 7 (7 to 8) mL/kg, 14 (14 to 16) breaths/minute and 106 (91 to 125) mL/kg/min, respectively. The median (IQR) time from initiation of mechanical ventilation to obtaining the initial arterial blood gas was 120 (37.5 to 180) minutes. Hypocapnia, normocapnia, and hypercapnia were found in 15% (11/75), 62% (47/75), and 23% (17/75) of patients, respectively.

**Figure 1:**
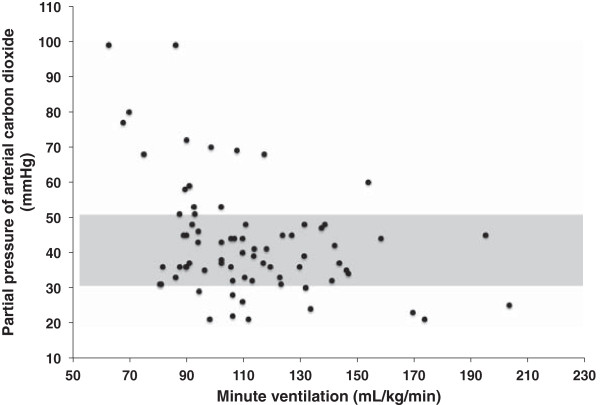
**Relationship between initial post-resuscitation prescribed minute ventilation and partial pressure of arterial carbon dioxide on initial arterial blood gas analysis after initiation of post-resuscitation ventilation settings.** Shaded area indicates range of normocapnia.

Figure 1 displays the relationship between initial post-ROSC prescribed MV and subsequent PaCO_2_. Initial post-resuscitation prescribed MV was found to have only a weak correlation with initial subsequent PaCO_2_, R = - 0.40 (*P* < 0.001). Using linear regression analysis we found R^2^ = 0.16, suggesting initial post-resuscitation MV is responsible for only 16% of the variability in subsequent PaCO_2_. Of note, prescribed RR alone was also found to have a weak correlation with initial subsequent PaCO_2_, R = - 0.26 (*P* = 0.02).

**Figure 2:**
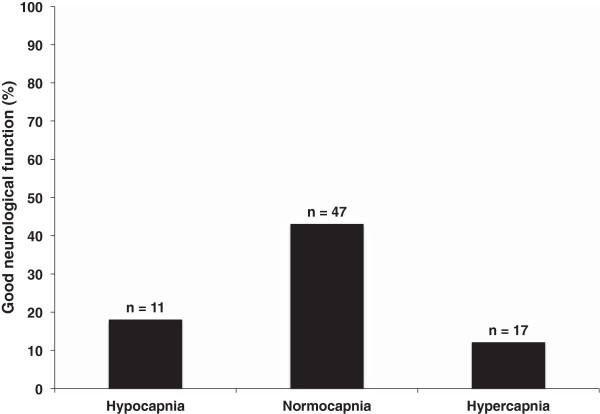
**Proportion of patients with good neurological function at hospital discharge (defined as a Cerebral Performance Category (CPC) 1 or 2) in relation to hypocapnia (PaCO**_**2**_ **≤ 30 mmHg), normocapnia (PaCO**_**2**_**31 to 49 mmHg), and hypercapnia (PaCO**_**2**_ **≥ 50 mmHg) on initial arterial blood gas analysis after initiation of post-ROSC ventilation settings.**

Thirty-two percent of all patients had good neurological function at hospital discharge. Figure 2 displays good neurological function at hospital discharge among patients with hypocapnia, normocapnia, and hypercapnia. Normocapnia was found to be associated with good neurological outcome on univariable analysis, odds ratio 4.44 (95%CI 1.33 to 14.85). Normocapnia remained associated with good neurological outcome on multiple sensitivity analyses (Table 3). A narrower PaCO_2_ range (between 35 and 45 mmHg) was not found to be associated with good neurological outcome 1.89 (95%CI 0.68 to 5.23).

**Table 3:** Results of sensitivity analyses: multivariable logistic regression models of the association between Normocapnia (defined as partial pressure of arterial carbon dioxide 31 to 49 mmHg) on initial post-resuscitation arterial blood gas, and good neurological function (defined as Cerebral Performance Category 1 or 2) at hospital discharge

**Variable**	**Beta**	**Standard error**	**Odds Ratio**	**95% LCI**	**95% UCI**	***P*-value**
Normocapnia	1.51	0.62	4.54	1.34	15.35	0.01
PEA/asystole initial rhythm	-0.65	0.58	0.52	0.17	1.63	0.26
Variable	Beta	Standard Error	Odds Ratio	95% LCI	95% UCI	*P*-value
Normocapnia	1.50	0.62	4.50	1.33	15.15	0.02
CPR > 20 minutes	-0.34	0.89	0.71	0.13	4.06	0.70
Variable	Beta	Standard Error	Odds Ratio	95% LCI	95% UCI	*P*-value
Normocapnia	1.24	0.63	3.46	1.01	11.89	0.04
Post-resuscitation shock^a^	-2.16	1.14	0.11	0.01	1.08	0.05
Variable	Beta	Standard Error	Odds Ratio	95% LCI	95% UCI	*P*-value
Normocapnia	1.36	0.63	3.89	1.14	13.26	0.03
Metabolic acidosis^b^	-0.76	0.54	0.47	0.16	1.34	0.16
Variable	Beta	Standard Error	Odds Ratio	95% LCI	95% UCI	*P*-value
Normocapnia	1.56	0.63	4.76	1.39	16.28	0.01
Age (decile)	-0.23	0.16	0.79	0.58	1.09	0.15
Variable	Beta	Standard Error	Odds Ratio	95% LCI	95% UCI	*P*-value
Normocapnia	1.49	0.62	4.44	1.31	15.11	0.02
Charlson comorbidity index	-0.44	0.28	0.64	0.37	1.10	0.11
Variable	Beta	Standard Error	Odds Ratio	95% LCI	95% UCI	*P*-value
Normocapnia	1.40	0.62	4.07	1.20	13.78	0.02
Pulmonary disease	-0.84	0.64	0.43	0.12	1.52	0.19
Variable	Beta	Standard Error	Odds Ratio	95% LCI	95% UCI	*P*-value
Normocapnia	1.41	0.62	4.11	1.21	13.93	0.02
Therapeutic hypothermia	-0.44	0.54	0.64	0.22	1.86	0.42

^a^Defined as systolic blood pressure < 100 mmHg or vasopressor support required to maintain systolic blood pressure > 100 mmHg during the first 24 hours after return of spontaneous circulation; ^b^Defined as a base deficit ≤ -6 during the first 24 hours after return of spontaneous circulation; CPR, cardiopulmonary resuscitation; LCI, lower confidence interval; PEA, pulseless electrical activity; UCI, upper confidence interval.

## Discussion

In this study, we found initial prescribed MV had only a weak correlation with subsequent PaCO_2_. We also found normocapnia on the initial ABG after initiation of post-ROSC ventilator settings was associated with good neurological function at hospital discharge. We believe these findings suggest (1) rapid achievement of normocapnia during the initial post-ROSC period could potentially improve neurological outcome and (2) given the prescribed MV cannot be used to reliably predict the subsequent PaCO_2_, early and multiple ABG analyses may be required during the initial post-ROSC period in order to achieve normocapnia.

We sought to test the correlation between initial post-ROSC prescribed MV and subsequent PaCO_2_ because given the association between PaCO_2_ derangements and poor outcome in different forms of brain injury [23,34-37], including post-cardiac arrest syndrome [5,6,38], we believe rapid achievement of normocapnia after ROSC could potentially attenuate the ongoing brain injury which occurs during post-cardiac arrest syndrome. The initial step towards developing a ventilation strategy aimed at achieving normocapnia immediately after ROSC, was to determine the relationship between initial post-ROSC prescribed MV and subsequent PaCO_2_.

After cardiac arrest many patients require mechanical ventilation. During mechanical ventilation the prescribed MV can be used to alter the PaCO_2_, which in turn can affect cerebral blood flow [13]. The prescribed MV is the product of the TV and the RR, and manipulation of either the TV or the RR will alter the MV and affect the PaCO_2_. In addition to identifying a weak correlation between initial prescribed MV and initial PaCO_2_, we also found a weak correlation between prescribed RR and initial PaCO_2_. The 2010 AHA Guidelines for Cardiopulmonary Resuscitation and Emergency Cardiovascular Care recommend that an initial TV of 6 to 8 mL/kg be prescribed after ROSC [13]. This recommendation was based on acute respiratory distress syndrome (ARDS) data, which found lower tidal volume was associated with a decrease in acute lung injury and improvement in clinical outcomes [22,39]. However, although lung injury has been demonstrated to occur in post-cardiac arrest syndrome the majority of patients die from neurological injury [16]. We suggest that a better post-cardiac arrest ventilation strategy would be a brain-centered approach aimed at preventing post-resuscitation PaCO_2_ derangements. Currently we are unaware of any data to support the use of a specific ventilation strategy in post-cardiac arrest syndrome. Specifically, there has been a paucity of data on the subject of initial prescribed MV and PaCO_2_ during the initial post-ROSC period. Although the AHA guidelines suggest titrating minute ventilation to achieve a PaCO_2_ 40 to 45 mmHg, they go on to state there are no data on specific ventilation strategies in post-cardiac arrest syndrome. To our knowledge this is the first report describing the relationship between initial prescribed MV and PaCO_2_ in post-cardiac arrest syndrome.

PaCO_2_ is a major regulator of cerebral blood flow after brain injury. Although it has been suggested that the degree of reactivity to PaCO_2_ may be blunted during the initial post-cardiac arrest period [40-42], recent literature suggests it remains intact [8,43,44]. Hypercapnia has been demonstrated to decrease cerebrovascular resistance and increase blood flow, suggesting a potential benefit in patients suffering from ischemic brain injury [8,45]. A recent registry study of post-cardiac arrest patients found no difference in survival to hospital discharge between hypercapnic and normocapnic patients overall; however, among survivors they found hypercapnic patients to have a greater likelihood of being discharged directly to home as opposed to a rehabilitation facility or transferred to another hospital [38]. Contrary to these findings, recent studies of traumatic brain injury and post-cardiac arrest syndrome in both pediatric and adult patients have demonstrated hypercapnia to be association with poor clinical outcomes [5,6,23]. The association between hypercapnia and poor outcome has been suggested to be secondary to hypercapnia-induced cerebral vasodilation and increased intracranial volume resulting in increased intracranial pressure and decreased cerebral perfusion, as well as compounding metabolic acidosis, which is common after ROSC [6,10-12]. However, both traumatic brain injury and pediatric cardiac arrest patients have important differences in physiological characteristics compared to adult cardiac arrest patients. It therefore remains unclear if hypercapnia is associated with poor neurological outcome in adult cardiac arrest patients.

Post-resuscitation hypocapnia has also been demonstrated to be associated with poor outcome [5,6,38]. In contrast to hypercapnia, the association between hypocapnia and poor outcome has been suggested to be secondary to hypocapnia-induced cerebral vasoconstriction resulting in decreased cerebral blood flow and increased cerebral ischemia potentially exacerbating anoxic brain injury [6-9].

We sought to perform this study because we believe developing an initial ventilation strategy to prevent PaCO_2_ derangements immediately after resuscitation from cardiac arrest could potentially allow for a therapeutic approach to help attenuate brain injury associated with post-cardiac arrest syndrome. In this observational study we answered several preliminary questions: (1) initial prescribed MV had only a weak correlation with subsequent PaCO_2_, and (2) early normocapnia was associated with good neurological outcome. Our findings suggest that the initial post-resuscitation PaCO_2_ is not solely the result of the prescribed MV, and thus it is likely influenced by other treatment-related factors (for example, use of neuromuscular blocking agents, duration and technique of bag ventilation by hand prior to initiation of mechanical ventilation) and multiple patient-related factors (for example, etiology of cardiac arrest, lung compliance, lung injury, intrinsic respiratory drive, persistent post-resuscitation circulatory shock, dead space, body habitus). These data provide scientific rationale for further research to determine the other factors associated with PaCO_2_ derangements during the initial post-ROSC period. Determining such factors will allow for the development of a volume-targeted ventilation strategy, which focuses on initial prescribed MV adjusted for individual patient-related factors. Such a ventilator strategy could potentially achieve normocapnia more consistently on the initial ABG after ROSC, decreasing the need for ventilator adjustments and additional ABG analyses, and ultimately decrease the time to achievement of normocapnia.

We acknowledge that this study has important limitations to consider. First, we found prescribed MV to have only a weak correlation with initial post-resuscitation PaCO_2_, suggesting PaCO_2_ is also influenced by patient-related factors; however, although this was a registry study of prospectively collected PaCO_2_ analyses and ventilator settings, patient-related factors were not initially collected and thus we were unable to determine what other factors were associated with initial post-resuscitation PaCO_2_. Many potentially important other factors are not routinely recorded in the patient record (for example, duration and technique of bag ventilation by hand prior to initiation of mechanical ventilation, lung compliance, lung injury, intrinsic respiratory drive, persistent post-resuscitation circulatory shock, dead space) and thus rigorous prospective research is required to determine the other determinates of initial post-resuscitation PaCO_2_. Second, this was an observational study and there exists the potential of unmeasured confounders (for example post-resuscitation lactic acidosis). Third, this study was limited to one center and it is possible that a study of larger scope could have found different results. Fourth, we defined hypocapnia and hypercapnia as PaCO_2_ ≤ 30 mmHg and PaCO_2_ ≥ 50 mmHg, respectively, based on PaCO_2_ levels from previously published studies [5,6,23]. We did not find a narrower PaCO_2_ range to be associated good neurological outcome suggesting severe PaCO_2_ derangements may be more detrimental to neurological outcome than smaller PaCO_2_ derangements; however, exact PaCO_2_ levels associated with good neurological outcome are unknown. Fifth, we did not perform any monitoring of physiological parameters related to PaCO_2_, such as transcranial Doppler monitoring. Sixth, it is important to consider that the majority of the patients in this study were in-hospital and PEA/asystole cardiac arrests, and these patients are known to have an exceptionally poor prognosis for survival. Lastly, this was an observational study and thus we can only report an association between normocapnia and good neurological outcome, rather than infer causation.

## Conclusions

In this sample of adult patients resuscitated from cardiac arrest, we found initial post-resuscitation prescribed MV had only a weak correlation with subsequent PaCO_2_. We also found early normocapnia was associated with good neurological function at hospital discharge. These data provide rationale for future research to determine the impact of PaCO_2_ management during mechanical ventilation in post-cardiac arrest patients.

## Abbreviations

ABG: arterial blood gas; AHA: American Heart Association; ARDS: acute respiratory distress syndrome; CPC: Cerebral Performance Category; CPR: cardiopulmonary resuscitation; IBW: ideal body weight; MV: minute ventilation; PaCO_2_: partial pressure of carbon dioxide; PEA: pulseless electrical activity; ROSC: return of spontaneous circulation; RR: respiratory rate; TV: tidal volume; VT: ventricular tachycardia; VF: ventricular fibrillation.

## Competing interests

The authors declare that they have no competing interests.

## Authors’ contributions

All authors have made substantial contributions to this paper: BWR supervised all aspects of the study and takes responsibility for the paper as a whole. BWR, ST, JHK, and MEC conceived this study. BWR and JHK acquired and managed the data. BWR and ST analyzed the data and interpreted results. BWR drafted the manuscript and all authors contributed substantially to its revision. All authors approved the manuscript in its final form.
